# Dietary Shifts in the Adaptation to Changing Marine Resources: Insights from a Decadal Study on Greater Lizardfish (*Saurida tumbil*) in the Beibu Gulf, South China Sea

**DOI:** 10.3390/ani14050798

**Published:** 2024-03-04

**Authors:** Xiaodong Yang, Yujian Deng, Jiao Qin, Konglan Luo, Bin Kang, Xiongbo He, Yunrong Yan

**Affiliations:** 1 Fisheries College, Guangdong Ocean University, Zhanjiang 524088, China; yangxd2832@163.com (X.Y.); yujiandeng@126.com (Y.D.); luokonlan@gmail.com (K.L.); 2 Southern Marine Science and Engineering Guangdong Laboratory, Zhanjiang 524000, China; qinjiao@zjblab.com; 3 Fisheries College, Ocean University of China, Qingdao 266003, China; kangbin@ouc.edu.cn; 4 Guangdong Provincial Engineering and Technology Research Center of Far Sea Fisheries Management and Fishing of South China Sea, Guangdong Ocean University, Zhanjiang 524088, China

**Keywords:** greater lizardfish, *Saurida tumbil*, dietary behavior, ontogenetic dietary shifts (ODSs), trophic levels, stable isotopes analysis, stomach content analysis

## Abstract

**Simple Summary:**

In this study, the author conducted a comparative analysis of Greater lizardfish (*Saurida tumbil*) in the Beibu Gulf during two distinct periods (2010 and 2020), aiming to comprehend the variations in dietary strategies and trophic levels while investigating the driving factors influencing *S. tumbil* in the study area. Over the past decade, the main prey items of this species have been fishes, followed by cephalopods and crustaceans. However, changes in the community structure and primary prey resources have led the *S. tumbil* population to diversify their prey species, utilize alternative resources, and expand their foraging space. The timing and magnitude of ODSs varied between the two periods. In comparison to 2010, the proportion of population feeding on pelagic–neritic prey significantly increased, and the δ^15^N values decreased, indicating that the shift in the ecological types of preferred prey from demersal to pelagic–neritic was the primary cause of the decrease in trophic levels.

**Abstract:**

Understanding dietary behavior during the individual development of marine predators and its temporal variations elucidates how species adapt to changes in marine resources. This is crucial for predicting marine predators’ habitat selection and the natural population’s responses to environmental changes. The authors conducted a comparative analysis of dietary shift strategies and trophic level variations in Greater lizardfish (*Saurida tumbil*) in the Beibu Gulf during two distinct periods (2010 and 2020) using stomach content and stable isotope analysis methods. Possible driving factors for these changes were also explored. Changes in the fishery community structure and the decline in the abundance of primary prey resources have led the *S*. *tumbil* population to diversify their prey species, utilize alternative resources, and expand their foraging space. However, the species’ foraging strategy, characterized by chasing and preying on schooling and pelagic prey, promoted stability in their feeding behavior across spatial and temporal scales. The main prey items remained demersal and pelagic fish species, followed by cephalopods and crustaceans. Similar to other generalist fish species, ontogenetic dietary shifts (ODSs) indicated a partial transition towards larger prey items. However, the timing and magnitude of the ODSs varied between the two periods, reflecting life-history variations and adaptive adjustments to environmental changes. In comparison to 2010, the population’s mean body length (BL) increased in 2020, and the proportion of the population feeding on pelagic–neritic prey significantly increased. However, the δ^15^N values were lower, indicating that the shift in the ecological niche of preferred prey from demersal to pelagic–neritic was the primary cause of the decrease in trophic levels. In the future, we will conduct further quantitative research integrating the spatiotemporal data of both predators and prey to clarify the relationships between marine predators’ feeding behavior, trophic levels, and changes in prey community structure.

## 1. Introduction

Understanding ontogenetic dietary shifts, the changes in diet utilization occurring over the life span of an individual consumer [1], related to trophic interactions provides fundamental insights into trophic theory [2,3]. The consequences of ODSs are acknowledged to exist at multiple levels, ranging from the individual to the ecosystem level [4,5,6]. Individual and population levels have received a lot of attention because ODSs usually have a positive effect on the growth rate, survival, and recruitment of many fish species [7], consequently influencing the lifetime fitness of individual fish and population dynamics [5,8], which is critical for promoting ecological knowledge and developing management plans for commercial species [9]. Quantifying trophic interactions in ontogenetic variation is important for both understanding life-history traits and elucidating how species’ ecological roles may be shaped by ontogeny [10].

Specifically, the top–down control exerted by certain marine predators, such as pinnipeds, cetaceans, tunas, and sharks, can significantly impact the abundance and dynamics of lower trophic levels in marine ecosystems [9,11,12,13,14]. In the last few decades, empirical and theoretical studies of marine predators’ ODSs have been conducted [15]. These studies have revealed the adaptability of ODSs as well as multiple patterns that occur throughout the life cycle of various marine predators [16]. In recent years, there has been an increase in interest in elucidating the driving mechanisms of ODSs, and a variety of determining factors, including some direct and indirect factors, have been proposed (e.g., predation risk, competition, prey availability and suitability, habitat use, and internal factors) [1]. Indeed, human activities and climate change can have a significant impact on these factors [17,18]. Under these disturbed conditions, comparative research on ODSs and the associated shifts for targeted species is critical for gaining a thorough understanding of the adaptive trophic roles of marine predators [19].

The overfishing of piscivorous predators, which alters the organization and structure of entire marine communities via a trophic cascade [20], has become a major issue for marine ecosystems worldwide [21]. Overfishing affects not only the life-history traits of marine species [22] but also their prey resources (e.g., forage fish species) [23]. Understanding these complex interactions between overfishing, fish populations, and prey resources remains a serious challenge [10,24]. However, it has recently been highlighted that the trophic niche shift of ODSs can aid in regulating fishing-induced dynamics of predator–prey dynamics, which plays an important role in understanding the resilience and recovery of overexploited marine populations [21,25].

The Beibu Gulf (17°–21.75° N, 105.67°–110.17° E) is a semi-closed bay in the northwestern South China Sea (SCS). This gulf has a relatively high level of fish diversity (about 960 species have been reported) and holds significant value for fisheries as one of China’s four major fishing grounds, owing to its subtropical and monsoonal climate, unique geomorphological features, and nutrient-abundant salts [26]. However, decades of overfishing have resulted in the collapse of the fish community in this region, leading to a marked decrease in the abundance of many demersal predators with high trophic levels [27]. Yet, it is unclear how the trophic levels changes in these piscivorous fishes respond to overfishing.

The Greater lizardfish (*Saurida tumbil*), belonging to the Synodontidae fish species, is a warm-water marine fish species distributed in the Indo-Pacific region, ranging from the Red Sea and Africa’s eastern coasts to the Arabian Sea, the Sea of Oman, and the Arabian Gulf, reaching South East Asia and Australia [28]. It is reported to be one of the most important commercial species in the countries along its geographical distribution [29], but its stocks have been heavily exploited in many regions over the last few decades [30]. Our previous study has identified certain traits shifts during the individual development process of *S. tumbil*, including a decreased body size and an earlier maturity, as well as a significantly decreased abundance caused by overfishing in the Beibu Gulf population [31]. Other studies have described the feeding habits of *S. tumbil*, a top demersal predator, in several different regions [32,33,34], and the results of these studies imply a trophic plasticity in that what they ate varied dramatically in the area. Yet, information on the ODSs of this species remains limited, with only our previous study providing relevant content [35].

Monitoring dietary behavior and trophic variations in overexploited species with a high commercial value is critical for stock and demersal community conservation as well as for improving the management of fisheries, particularly in a highly exploited area like the South China Sea. Therefore, the objectives of this study were to investigate the population of *S. tumbil* in the Beibu Gulf during two periods (2010 and 2020), with a particular focus on (i) the shifts in dietary strategies occurring with temporal variations and individual development, and (ii) how these shifts affected the trophic level of *S. tumbil*.

## 2. Materials and Methods

### 2.1. Specimen Collection and Processing

**Figure 1:**
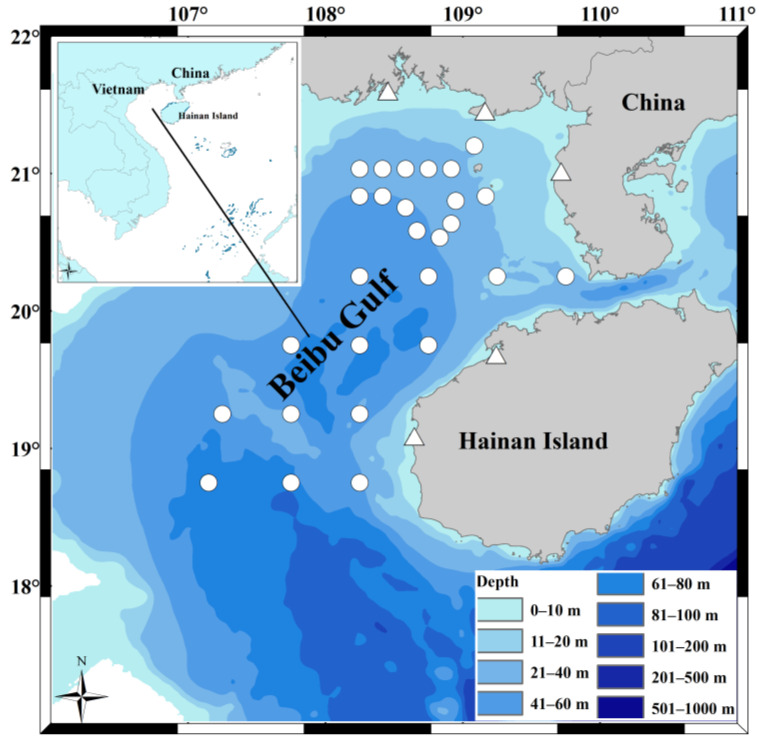
Sampling locations for the study of *Saurida tumbil* diets in the Beibu Gulf in 2010 and 2020. The symbols represent sites from different years; the white circles correspond to the specimen collection sites in 2010, while the white triangles correspond to the fishing vessels’ locations for specimen collection in 2020 at five fishing ports (Qisha, Beihai, Jianghong, Baimajing, and Basuo). This map was created using software ArcGIS v. 10.4.1.

Specimens of *S. tumbil* were collected from the Beibu Gulf in 2010 and 2020 using bottom trawler nets (maximum mesh size of 50 mm). In 2010, specimens were collected at 24 offshore locations using bottom trawler nets across four seasons (February, May, August, and November). In 2020, specimens were collected monthly from fishing vessels at five Chinese fishing ports around the Beibu Gulf (Figure 1). The local fishing vessels move inshore–offshore, except for the fishing moratorium (Notice of the Ministry of Agriculture (2013) 3 of the Republic of China). To facilitate a meaningful comparison of sample data between the two periods, we selected the specimens collected in 2020 during February, May, August, and November. For the stations (2010) or fishing vessels (2020) with high numbers of captured *S. tumbil*, some samples of different sizes were selected to ensure that the collected samples were representative in terms of time and space. However, because there were fewer *S. tumbil* caught with body lengths (BL, cm) longer than 20.0 cm (especially samples with BLs longer than 23.0 cm), all the experimental samples collected in this study were preserved.

To prevent the digestion of stomach contents, the collected specimens were immediately frozen at −20 °C and transported to the laboratory. The BL and wet weight (WW g) of each specimen were recorded. The collected samples were dissected one by one. During dissection, the individual’s body weight, gonad weight, and stomach contents weight were measured. The vacuity index (VC) was calculated using the following formula: VC = (empty stomachs/total stomachs) × 100. The gonad somatic index (GSI) of each specimen was calculated using the following formula: GSI% = (gonad weight/body weight) × 100 [36]. Additionally, the repletion index (RI) was calculated as follows: RI% = (total stomach contents weight/total fish weight) × 100. GSI% represents the developmental stage, while RI% reflects the feeding intensity. The stomach contents were analyzed under a stereomicroscope, and each prey item at the lowest possible taxonomic level was identified, counted, and weighed.

To quantitatively compare and evaluate dietary variations between the two periods, all the specimens were divided into five size classes with a 3 cm interval increment based on BL. The first class (I) included all immature specimens (<14.1 cm), the second class (II) included specimens with a BL between 14.1 and 17.0 cm, and the third class (III) contained individuals with a BL between 17.1 and 20.0 cm. The largest individuals (>20.0 cm) were grouped into two classes: IV (20.1–23.0 cm of BL) and V (BL > 23.0 cm).

To evaluate the contribution of each prey item, the following parameters were calculated: the percentage of abundance composition (N%), the percentage of biomass composition (W%), and the frequency of occurrence (F%) [37]. These indices are necessary to calculate the relative importance index, $\mathrm{IRI}=F\%\times \left(N\%+w\%\right)$, expressed as IRI% ($\mathrm{IRI}\%=\left(\mathrm{IRI}/\sum \mathrm{IRI}\right)\times 100$) [38].

The total niche width (TNW) of a population is defined as the variance in resource utilization across all individuals. It can be further divided into two components: within-individual variation (WIC) and between-individual variation (BIC) [39]. The degree of individual diet specialization can be quantified by the ratio of WIC to TNW (WIC/TNW), which ranges from 0 to 1. A smaller WIC/TNW ratio indicates a lower degree of ecological overlap between individuals and a higher level of specialization [40]. The proportional similarity index of diet (*PS_i_*) represents the average similarity in diet between pairs of individuals within a population [41]. The R package “RInSp” [42] was used for the calculation of TNW, WIC, BIC, WIC/TNW, and *PS_i_*.

### 2.2. Stable Isotopes Analysis (SIA)

For the SIA, specimens of dorsal muscle tissue were taken from each individual and preserved without any chemicals at −20 °C [43]. The muscle specimens were subjected to freeze-drying, carried out using a freeze-dryer at −55 °C for 48 h (Alpha1-4/2-4LD Plus, Christ, Osterode, Germany). The muscle specimens were then homogenized into a powder using a steel bead homogenizer (MiniBeadbeater-16, Biospec, Bangor, Pennsylvania, USA). In total, 176 and 277 muscle specimens were used for SIA analysis in 2010 and 2020, respectively. Approximately 0.40 mg of powder was weighed on a Mettler Toledo microscale for each specimen and packed. 

The specimens were tested using an elemental analyzer (EA IsoLink, Thermo Fisher scientific, Waltham, Massachusetts, USA) linked to an isotope ratio mass spectrometer (Thermo Scientific 253 Plus, Thermo Fisher scientific, Waltham, Massachusetts, USA) at Guangdong Ocean University, China. All stable isotope values are reported in the δ notation: $\left(\frac{R_{sample}}{R_{standard}}-1\right)\times 10^{3}$. In this notation, X is δ^15^N, R represents the ^15^N/^14^N ratio, and the standard is atmospheric nitrogen.

### 2.3. Data Treatment

A within-group analysis of variance (ANOVA) was conducted for size classes I, II, III, IV, and V to assess the differences in GSI%, RI%, and δ^15^N. Subsequently, within-group multiple comparisons were performed using Tukey’s Honestly Significant Difference (HSD) test. Paired *t*-tests were employed to compare data from 2010 to 2020.

To enhance the interpretability of the compositional matrix of food composition (IRI%), a square root transformation was applied to different categories. The Bray–Curtis similarity was calculated, and a clustering tree was constructed using the unweighted pair group method with an arithmetic mean (UPGMA) based on Bray–Curtis distances. The quality of the clustering results was evaluated using a Shepard plot, and the optimal number of clusters was determined based on the fusion level value plot.

Non-metric multidimensional scaling (nMDS) was utilized to ordinate the compositional matrix of food composition, enabling the observation of changes in food composition across individual developmental stages. A one-way ANOVA was performed to assess the differences in dietary composition between the size classes.

To quantify linear and polynomial trends, least squares regression was performed, and the δ^15^N values were plotted against the BL. The selection of the best-fitting line for the δ^15^N values was based on significant improvements in the *R*^2^ values and the *F*-values.

All statistical analyses were carried out using the software R v. 4.2.1 [44].

## 3. Results

### 3.1. Population Structure and Indices

In 2010 and 2020, a total of 670 and 708 specimens of *S. tumbil* with vacuity indices of 49.55% and 52.54%, respectively, were collected. Among these specimens, 338 and 336 had a mean BL of 17.20 ± 2.96 cm and 18.50 ± 2.89 cm, respectively (Table 1).

**Figure 2:**
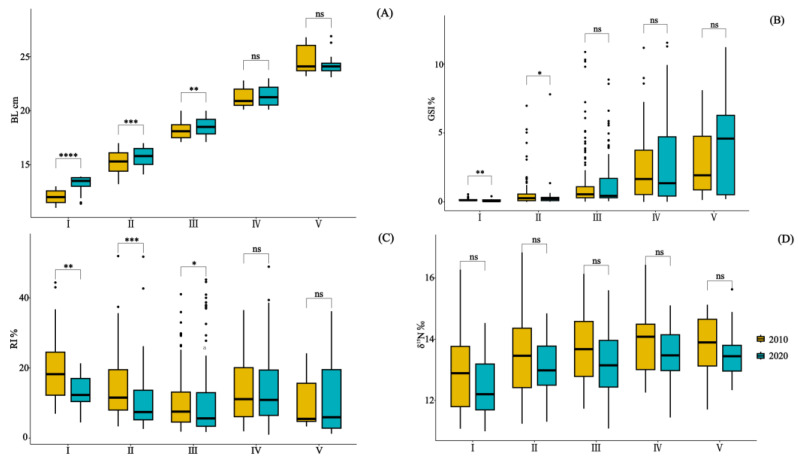
Illustrates the paired comparison of BL (**A**), GSI% (**B**), RI% (**C**), and δ^15^N values (**D**) in the stomach content specimens of *Saurida tumbil* captured in the Beibu Gulf in 2010 and 2020. I–V represents five size classes that scale the body length (BL): I (BL < 14.1 cm), II (BL = 14.1–17.0 cm), III (BL = 17.1–20.0 cm), IV (BL = 20.1–23.0 cm), and V (BL > 23.0 cm). GSI% (gonadosomatic index), RI% (relative index), and δ^15^N (nitrogen stable isotope values). * = *p* ≤ 0.05; ** = *p* ≤ 0.01; *** = *p* ≤ 0.001; **** = *p* ≤ 0.0001; and ns = *p* > 0.05.

The one-way analysis of variance (ANOVA) and subsequent post hoc multiple comparisons demonstrated significant variations in the GSI% and RI% among the size classes (*p* < 0.05) (Table 2). Furthermore, the paired *t*-tests conducted between the size classes indicated significant differences in the BL, GSI%, and RI% during the early developmental stages (I–III) (*p* < 0.05) (Figure 2).

### 3.2. Temporal Variation in Diet Composition and Types

Our stomach content analysis revealed that, in 2010, 34 prey species were identified, representing 19 families across three categories. In 2020, there were 58 prey species, belonging to 23 families across the same three categories. The primary prey category consisted of fish (IRI%_2010_ = 98.75%, F%_2010_ = 88.04; IRI%_2020_ = 95.24%, F%_2020_ = 82.61), followed by cephalopods (IRI%_2010_ = 1.08; IRI%_2020_ = 4.63) and crustaceans (IRI%_2010_ = 0.17; IRI_2_%_020_ = 0.12) (Table 3). These findings indicate that, over the past decade, there has been an increase in dietary prey diversity, a decrease in the proportion of fish consumption, and an increase in the proportion of cephalopod consumption.

In 2010, the dominant prey species (IRI% > 3) were primarily demersal species such as *Leiognathus lineolatus* (IRI% = 26.88), *Photopectoralis bindus* (IRI% = 15.74), and *Acropoma japonicum* (IRI% = 7.58) and pelagic–neritic species such as *Trachurus japonicus* (IRI% = 20.72), *Stolephorus indicus* (IRI% = 6.12), *Stolephorus* sp. (IRI% = 5.26), *Decapterus maruadsi* (IRI% = 4.59), *Loligo* sp. (IRI% = 4.04), and *Sardinella jussieu* (IRI% = 3.32) (see Table 3). In 2020, the dominant prey species (IRI% > 3) included demersal species like *Secutor ruconius* (IRI% = 4.31) and *Leiognathus* sp. (IRI% = 4.17) as well as pelagic–neritic species such as *Stolephorus* sp. (IRI% = 50.41), *Loligo* sp. (IRI% = 15.23), and *Sardinella lemuru* (IRI% = 5.95) (Table 3). Over the past decade, there has been a decrease in the number of dominant prey items, and the ecological composition has shifted from demersal to pelagic–neritic. Notably, with the exception of *A. japonicum*, all other dominant prey items exhibit schooling behaviors.

### 3.3. Variations in Diet Composition with Individual Development

**Figure 3:**
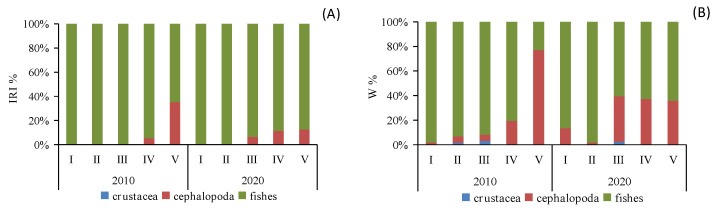
Composition IRI% (**A**) and W% (**B**) of the diet of different size classes of *Saurida tumbil* in 2010 and 2020. I–V represents five size classes that scale the body length (BL): I (BL < 14.1 cm), II (BL = 14.1–17.0 cm), III (BL = 17.1–20.0 cm), IV (BL = 20.1–23.0 cm), and V (BL > 23.0 cm). W% (percentage in biomass), IRI% (index of relative importance expressed as a percentage).

When analyzing the prey preferences across different size classes, it became evident that classes I and II (BL < 17.1 cm) primarily favored fish consumption, whereas classes III–V (BL > 17.0 cm) displayed an increasing preference for cephalopod consumption. This trend was especially prominent in class V, where cephalopod consumption saw a significant rise (IRI%_2010_ = 35.27%, W%_2010_ = 77.03%; IRI%_2020_ = 12.48%, W%_2020_ = 35.92%). Furthermore, class III exhibited the highest proportion of crustacean consumption (Figure 3).

**Figure 4:**
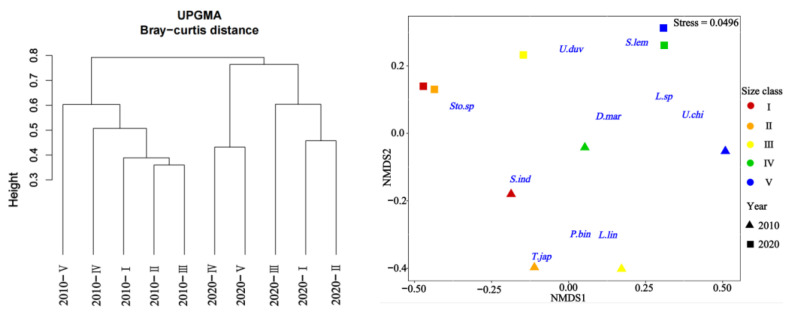
Tree clustering and nMDS sorting based on Bray–Curtis distance were performed using square root-transformed IRI% data to differentiate distinct size classes of *Saurida tumbil* in 2010 and 2020. I–V represent five size classes based on body length (BL): I (BL < 14.1 cm), II (BL = 14.1–17.0 cm), III (BL = 17.1–20.0 cm), IV (BL = 20.1–23.0 cm), and V (BL > 23.0 cm). *Sto.sp* = *Stolephorus* sp., *U.duv* = *Uroteuthis duvauceli*, *S.lem* = *Sardinella lemuru*, *D.mar* = *Decapterus maruadsi*, *Lei.sp* = *Leiognathus* sp., *U.chi* = *Uroteuthis chinensis*, *S.ind* = *Stolephorus indicus*, *T.jap* = *Trachurus japonicus*, *P.bin* = *Photopectoralis bindus*, and *L.lin* = *leiognathus lineolatus*.

**Figure 5:**
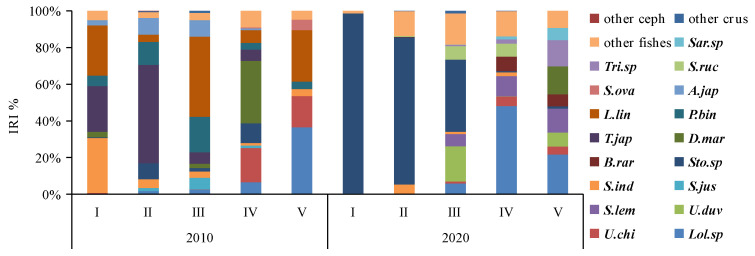
Changes in the dominant prey species (IRI%) consumed by different size classes of the *Saurida tumbil* in 2010 and 2020. I–V represent the five size classes based on body length (BL): I (BL < 14.1 cm), II (BL = 14.1–17.0 cm), III (BL = 17.1–20.0 cm), IV (BL = 20.1–23.0 cm), and V (BL > 23.0 cm). IRI% (index of relative importance expressed as a percentage). Other ceph = other cephalopods, other crus = other crustacea, *Sar.sp* = *Saurida* sp., *Tri.sp* = *Trichiurus* sp., *S.ruc* = *Secutor ruconius*, *S.ova* = *Solea ovata*, *A.jap* = *Acropoma japonicum*, *L.lin* = *leiognathus lineolatus*, *P.bin* = *Photopectoralis bindus*, *T.jap* = *Trachurus japonicus*, *D.mar* = *Decapterus maruadsi*, *B.rar* = *Bregmaceros rarisquamosus*, *Sto.sp* = *Stolephorus* sp., *S.ind* = *Stolephorus indicus*, *S.jus* = *Sardinella jussieu*, *S.lem* = *Sardinella lemuru*, *U.duv* = *Uroteuthis duvauceli*, *U.chi* = *Uroteuthis chinensis*, and *Lol.sp* = *Loligo* sp.

Cluster analysis and MDS ordination were conducted on the prey composition matrix (IRI%) based on the size classes. In 2010, the specimens were divided into two distinct categories (Figure 4). The first category encompassed specimens from classes I, II, III, and IV, sharing an IRI% similarity of over 55%. Among these classes, the main prey for classes I-III included species like *P. bindus*, *L. lineolatus*, *A. japonicum*, *S. indicus*, *Stolephorus* sp., *T. japonicus*, and *S. jussieu*, among others. Class IV also exhibited some consumption of *Stolephorus* sp., *T. japonicus*, and *L. lineolatus*, but its primary prey items were *D. maruadsi* and cephalopods. The second category consisted solely of specimens from class V, which exhibited a distinct preference for cephalopods and *L. lineolatus*, as depicted in Figure 5. Furthermore, our one-way ANOVA analysis demonstrated variations in dietary composition among the *S. tumbil* size classes (*p* < 0.05). Class V demonstrated marked distinctions from all other size classes, while no statistically significant differences (*p* > 0.05) were observed in the dominant prey species among classes I, II, III, and IV.

In 2020, the specimens were divided into three distinct categories (Figure 4). The first category comprised specimens from classes I and II, exhibiting a high IRI% similarity of 82%. Within this category, the *Stolephorus* sp. emerged as the most significant prey, with IRI% values ranging from 80.29% to 98.08%. The second category exclusively included specimens from class III that displayed an increased consumption of species such as *S. lemuru*, *Bregmaceros rarisquamosus*, *S. ruconius*, and *Loligo* sp. The third category encompassed specimens from classes IV and V showing an IRI% similarity of 53%. In this category, the *Loligo* sp. dominated as the primary prey, while consumption of *D. maruadsi*, *Trichiurus* sp., *Saurida* sp., and other species was also observed (Figure 5). Our one-way ANOVA analysis demonstrated significant differences (*p* < 0.001) between classes I and II in comparison to classes IV and V. Additionally, it indicated significant differences (*p* < 0.001) between class III and the other classes. These findings emphasize the distinct prey consumption patterns among the size classes in 2020.

**Figure 6:**
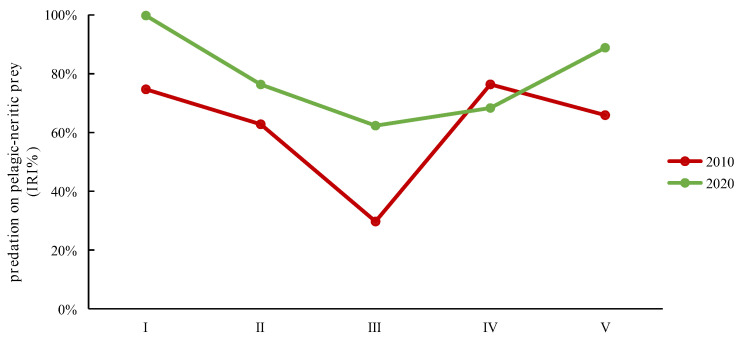
The IRI% variations in predation on pelagic–neritic prey by different size classes of *Saurida tumbil* in 2010 and 2020. I–V represent the five size classes based on body length (BL): I (BL < 14.1 cm), II (BL = 14.1–17.0 cm), III (BL = 17.1–20.0 cm), IV (BL = 20.1–23.0 cm), and V (BL > 23.0 cm). IRI% (index of relative importance expressed as a percentage).

Analyzing the different size classes over the two time periods in terms of the ecology of their types of prey revealed distinct patterns. In both 2010 and 2020, classes I, II, IV, and V exhibited a primary preference for pelagic–neritic prey. These classes consistently consumed a substantial proportion of pelagic–neritic prey. However, the proportion of pelagic–neritic prey consumed by class III significantly decreased. Furthermore, in 2020, the overall proportion of pelagic–neritic prey consumption was notably higher compared to 2010, with the exception of class IV (Figure 6). These observations emphasize the dynamic nature of prey selection among the different size classes over the two time periods, highlighting variations in their ecological roles and preferences.

### 3.4. Individual Specialization and Trophic Niche Widths

Based on the prey abundance data found during our stomach contents’ analysis, an investigation into the population’s trophic niche was conducted. It was observed that, as individuals developed, there was an increase in within-individual resource utilization differences (WIC), leading to a rise in the WIC/TNW ratio and a concurrent decrease in dietary specialization. The total niche width (TNW) of the different size classes initially exhibited an upward trend, followed by a subsequent decline. When comparing the population’s trophic niche between the two distinct time periods, it was observed that, in 2020, the population exhibited a higher TNW, while the WIC/TNW ratio was lower (Table 4).

Pearson’s correlation analysis revealed a negative correlation between TNW and WIC/TNW (*R*^2^ = −0.77), as well as between TNW and *PS_i_* (a measure of dietary specialization) (*R*^2^ = −0.96), in 2010. In contrast, in 2020, TNW displayed a positive correlation with WIC/TNW (*R*^2^ = 0.54) and a negative correlation with *PS_i_* (*R*^2^ = −0.98).

### 3.5. Variations in Trophic Levels

**Figure 7:**
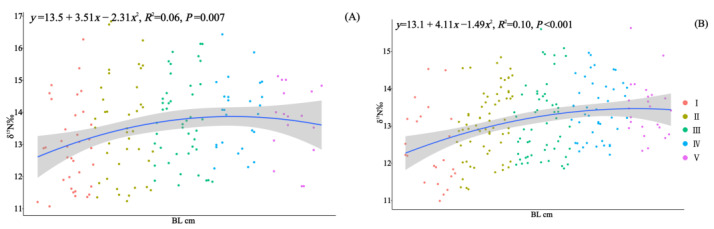
Correlation analysis of body length (BL, cm) and δ^15^N for *S. tumbil* captured in 2010 (**A**) and 2020 (**B**). I–V represent the five size classes based on body length (BL): I (BL < 14.1 cm), II (BL = 14.1–17.0 cm), III (BL = 17.1–20.0 cm), IV (BL = 20.1–23.0 cm), and V (BL > 23.0 cm). The line represents the linear regression line, and the shadow represents the standard error interval of the regression line. δ^15^N (nitrogen stable isotope values).

In 2010 and 2020, 164 and 210 specimens of *Saurida tumbil* were randomly selected, respectively, for stable isotope nitrogen analysis. Our one-way analysis of variance results revealed significant differences in the δ^15^N values among the different size classes in both 2010 (*F* = 2.805, *p* < 0.02) and 2020 (*F* = 5.408, *p* < 0.001) (Table 2). The δ^15^N values exhibited an increasing trend with individual development (Figure 7). Pairwise *t*-tests indicated that there were no significant differences (*p* > 0.05) in the δ^15^N values among the size classes between the two time periods. Furthermore, compared to 2010, the mean δ^15^N values for each size class showed a decrease in 2020 (Table 2). 

## 4. Discussion

Understanding the dietary behavior of individual predators throughout their developmental process and the temporal variations in this behavior is of paramount importance for elucidating intraspecific changes in the ecological roles of species and how populations adapt to fluctuations in the availability of marine resources [45,46,47]. This information is indispensable for predicting the habitat selection of marine predators and the responses of natural populations to environmental changes [48]. In this study, the authors conducted a comparative analysis of the dietary strategies and trophic levels of *S. tumbil* in the Beibu Gulf during two distinct periods (2010 and 2020).

### 4.1. Temporal Shifts in Dietary Strategies

Over the past decade, the primary prey of *S. tumbil* in the Beibu Gulf has consistently been fish, followed by cephalopods and crustaceans. This pattern aligns with the findings of several studies [33,34] as well as similar research outlining fish, mollusks, and crustaceans as the main prey items [49]. These findings underscore the carnivorous dietary behavior and stable dietary preferences of *S. tumbil* across various spatial and temporal scales. While there exists diversity in the types of prey and variations in the proportions of different prey types, it is noteworthy that, for both periods under investigation, the predominant prey type primarily consisted of fish and cephalopods who often exhibit clustering or migratory behaviors. This observation lends support to the conclusion that *S. tumbil* is adept at actively pursuing prey over significant distances, occasionally engaging in short-distance vertical migration [50]. Consequently, it appears that, in most instances, *S. tumbil* opts for a mobile foraging strategy when targeting prey in the demersal and pelagic–neritic water column, rather than adopting a stationary approach for benthic prey.

Furthermore, this rapid pursuit of swimming prey exhibits remarkable consistency and stability over varying time scales. Given that foraging activities typically involve considerable energy expenditure, predators may employ different strategies, differing by foraging time and effort, search methods, and prey preferences, to minimize energy costs within a specific habitat [51,52]. When a particular dietary strategy yields greater returns, it is likely to be repeated over time, fostering the development of behavioral consistency [53]. This phenomenon of consistent dietary behavior has been observed in diverse marine animal populations, particularly among the top marine predators [54,55]. Clustering or migratory behaviors have also been shown to promote the development of behavioral consistency within a population [56]. Therefore, we posit that *S. tumbil*’s rapid horizontal and vertical pursuit of schooling prey significantly contributes to the stability of its dietary composition across spatial and temporal scales.

Additionally, the authors noted an increase in the diversity of prey species within the diet of *S. tumbil* from 34 species to 58 species. Among these prey species, 28 were consistently present in the diet items of both time periods, while the species exclusive to one time period mostly shared family affiliations with the 28 common prey species, with some belonging to benthic fish families. This observation suggests that fish species within the same family can serve as alternative prey for *S. tumbil* due to their shared biological characteristics and ecological similarities, whereas specialized benthic fish species utilize specific habitat niches. These changes in dietary diversity are also reflected in the dietary niche index. When comparing 2010 (TNW = 2.737) with 2020 (TNW = 3.267), a significant increase in the total niche width of the population became evident. However, the individual diet specialization index WIC/TNW did not exhibit significant changes (0.039 in 2010, 0.036 in 2020), while the dietary similarity coefficient *PS_i_* noticeably decreased (0.117 in 2010, 0.088 in 2020). These shifts in prey composition and dietary niche can be attributed to alterations in individual biological traits as well as changes in prey resource abundance, availability, and diversity. It has been suggested that factors such as age, gender, and reproductive status can influence individual variations in dietary behavior [56]. Manojkumar et al. [33] found that changes in the diet of *S. tumbil* are linked to seasonal fluctuations in resources, the presence of certain species’ juveniles, and fish migration. In this study, variations in food composition among *S. tumbil* populations with a BL below 20.1 cm during the two time periods could be attributed to differences in population development and feeding intensity. However, the primary driving factor is likely to be the changes in prey resources.

The dominant fish species in the Beibu Gulf area (IRI% > 5%) decreased from seven species in 2011 (*L. lineolatus*, *A. japonicum*, *S. tumbil*, *Evynnis cardinalis*, *T. japonicus*, *D. maruadsi*, and *P. bindus*) to three species in 2018 (*E. cardinalis*, *A. japonicum*, and *T. japonicus*). Among these, the most significant decline was observed in *D. maruadsi*, transitioning from a dominant to a rare species [57], reflecting a decrease in the diversity and abundance of accessible prey in the diet of *S. tumbil*. To alleviate resource competition among different developmental groups and meet energy demands during individual development, *S. tumbil* might opt for same-family species with similar biological and ecological characteristics as alternative prey resources throughout its life stages or during shifts in individual dietary preferences. This may include species such as *S. lemuru* from the Clupeidae, *Nuchequula nuchalis*, *Leiognathus berbis*, and *Gazza minuta* from the Leiognathidae, as well as *Ostorhinchus gularis*, *Jaydia carinatus*, and *Rhabdamia gracilis* from the Apogonidae. Additionally, they might consume some benthic fish with limited swimming abilities, such as *Oxyurichthys tentacularis*, *Trypauchen vagina*, *Oxyurichthys* sp., *Brachypleura novaezeelandiae*, *Pterygotrigla hemisticta*, and *Solea ovata*. This strategy of diversifying the resource base and expanding feeding space may have contributed to the expansion of the habitat distribution and ecological feeding niche of *S. tumbil*, enabling it to adapt to changing ecological environments and sustain population growth.

### 4.2. Shifts in Dietary Strategies throughout Individual Development

The highest proportion among fish prey is comprised of small-sized fish from families like Gobiidae and Belonidae, followed by medium-sized fish and cephalopods. During the individual developmental process, there is a decrease in the consumption ratio of small fish and an increase in the consumption ratio of medium-sized fish and cephalopods. Moreover, across different size classes, they also exhibit resource-sharing behaviors, as exemplified by preying on species like *S. indicus*, *B. rarisquamosus*, *T. japonicus*, and *D. maruadsi*. Studies conducted in various marine regions have consistently documented substantial shifts in prey composition during the growth stages of *S. tumbil*, accompanied by a statistically significant positive correlation (*p* < 0.05) between prey size and predator size [33,50]. These observations suggest that individuals of the *S. tumbil* species, a generalist species, undergo partial shifts in their prey selection toward larger prey during their development, in contrast to specialist species [58], which exhibit complete shifts. This implies that, akin to many predators, *S. tumbil* undergoes ODSs (Ontogenetic Diet Shifts) strategies due to changes in individual condition, energy requirements, habitat space, and resource structure [59,60,61]. Such dietary shifts accommodate early developmental needs while facilitating the more efficient utilization of food resources by later-stage individuals, thereby conferring a competitive advantage in food competition [62]. These findings align with the “optimal foraging theory”, which posits that, in their pursuit of maximizing energy acquisition while minimizing expenditure, fish tend to preferentially select larger prey during their feeding activities [63].

Due to fluctuations in prey diversity and prey abundance as well as a shift towards squid consumption, the 2010 population was classified into two groups. In contrast, the 2020 population was categorized into three groups. In comparison to the 2010 population, the 2020 population exhibited a decrease in the number of dominant prey fish species across various developmental stages. However, there was a noticeable advancement in the developmental stage at which cephalopods became primary prey (BL > 17.0 cm). This indicates that the timing and extent of the dietary transition during an individual’s development differed between the two time periods. In most animal species, there is a shift in individual development and ecological niche during complex life cycles [64]. The variations in trophic interactions and the transition in individual development are closely linked to changes in energy requirements and are influenced by factors such as the life history, environmental conditions, prey resources, and the structure of the food web [65,66,67]. The mean BL values of specimens from the 2020 population and each size class exceeded those of the 2010 population, and significant differences in BL and GSI% were observed when the BL was less than 20.0 cm. Therefore, we infer that the changes in energy requirements and behavioral patterns associated with individual status differences may be one of the key factors contributing to the variation in the timing of dietary shifts. The differences in the dominant prey species and their abundance during individual development between the two periods may be attributed to fluctuations in resources [68].

Yan et al. [69] discovered that, in the prey items of *S. tumbil*, aside from *Stolephorus* sp. and *T. japonicus*, other species exhibited significant fluctuations, with most prey appearing only occasionally in single months. The dietary behavior of *S. tumbil* displayed a degree of random selectivity. Over time, studies have demonstrated that changes in the habitat environment and prey resources in the Beibu Gulf have occurred due to climate change, human disturbance, and related food web disruptions [70,71]. As a representative generalist species, *S. tumbil* individuals exhibits partial dietary shifts during their development, and alternative prey resources become available during this period [6,10].

Changes in dietary strategies during an individual’s development are also evident in terms of dietary specialization and trophic niche. As individuals develop, a decrease in population-level specialization is accompanied by an increase in dietary similarity indices. It is postulated that the early developmental stages bring about genetic differences that result in variations in feeding capability and efficiency. As individuals develop, an advantage in feeding ability stemming from an increased body size leads to higher growth rates and greater access to a broader range of food resources. However, in 2020, size classes I and II exhibited a significant shift towards predominantly consuming *Stolephorus*.sp as prey (IRI% range 80.29–98.08%), leading to lower specialization and higher dietary similarity. This shift may be attributed to within-habitat variations in the abundance and availability of prey resources within the habitat.

In 2010 and 2020, respectively, class IV and class III exhibited relatively broader trophic niches compared to other groups, as indicated by our dietary composition analysis. These groups not only preyed on small- and medium-sized fish and cephalopods but also had a notably higher proportion of crustaceans in their diet compared to other groups. This suggests that class III or IV might be competitively positioned between other size classes. To alleviate the intensity of dietary competition and meet energy requirements during individuals’ development, the individuals within these classes have increased their utilization of the benthic habitat’s space and resources over the years. Simultaneously, these individuals may compete with those of other size classes for specific resources, thus demonstrating a potentially more advantageous dietary strategy. Similar observations were made by Xia [72] in a study of *Megalobrama terminalis*’ ODSs.

### 4.3. Variations in Trophic Levels

Based on nitrogen stable isotope analysis, it was observed that both populations of *S. tumbil* exhibited shifts in their trophic level during an individual’s development; however, these shifts were not consistent. BL emerged as a significant predictor of δ^15^N values, with enrichment occurring as individuals increased in size. Similar to other apex predators, as *S. tumbil* grows, factors such as swimming speed, gape width, and energy requirements increase, resulting in an elevation in their trophic level within the corresponding food web [61,73]. In 2010, δ^15^N enrichment was observed within individuals with a BL below 23.0 cm (class I–IV), with the transition in trophic level occurring between class III and class IV. In 2020, δ^15^N continued to exhibit enrichment across the developmental stages, with a trophic status transition occurring between class I and II. There incongruent shifts in trophic level can be elucidated by the results obtained from our analysis of diet composition, which was based on individuals’ stomach contents. In 2010, the most notable change in classes III and IV was an increase in the proportion of squid consumed, whereas, in classes I and II, the most significant change was an increase in the number of prey species consumed. The increase in prey species or a shift in prey type led to alterations in the enrichment of δ^15^N values.

The mean BL for each size class was higher in 2020 than in 2010, but the mean δ^15^N values were lower in 2020. This suggests an overall decrease in the trophic level of Beibu Gulf *S. tumbil* over the past decade. After combining the results of our stomach content analyses, it became evident that, in 2020, the population not only displayed a reduced preference for fish prey species but also shifted their preference from demersal prey (such as *l. lineolatus*, *P. bindus*, and *A. japonicum*) to pelagic–neritic prey (such as *Stolephorus* sp.). It is hypothesized that this dietary shift is the primary cause of the decreased trophic level observed in *S. tumbil* in the Beibu Gulf.

Previous studies have indicated that, since 1984, intensive fishing has led to a shift in Chinese marine catch from long-lived, high-trophic-level benthic fish to short-lived, low-trophic-level invertebrates and pelagic–neritic fish. This shift has caused the marine trophic index (MTL), which measures the change in the mean trophic level, to fall below the global average. Fish are the main contributors to the trophic index (with their contributions ranging from 73.1% to 85.8%) [74]. Over the past few decades, a severe decline in fishery resources due to overfishing, unsustainable development and utilization, and natural environmental changes in the Beibu Gulf has been observed [70,71,75]. Between 1961 and 2017, dominant species also underwent a transition from benthic to pelagic–neritic, going from a high trophic level to a low trophic level, with the MTL decreasing at a rate of 0.04 trophic levels per decade [27]. This indicates that over the past few decades, the overall food chain length and nutritional levels of consumers in the Beibu Gulf have been consistently degrading. *S. tumbil*, as a carnivorous and omnivorous consumer, occupies a high trophic level (TL ranging from 4.2 to 4.4) in the marine food web. Their response to changes in fishing community structure and prey resource availability has led to a decrease in trophic levels through ontogenetic diet shift (ODSs) strategies.

## 5. Conclusions

The results of this study indicate that, despite the *S. tumbil* in the Beibu Gulf employing foraging strategies involving rapid horizontal and vertical movements to pursue and capture pelagic–neritic prey, promoting stability in its dietary behavior across spatial and temporal scales, the primary prey types remain benthic and pelagic–neritic fishes, followed by cephalopods and crustaceans, consistent with the findings of numerous other studies. However, against the backdrop of overfishing, significant shifts in the population’s dietary composition have occurred with temporal variation and during individuals’ development, leading to subsequent alterations in the trophic levels.

The changes in the structure of the fishery community and the decline in the abundance of dominant fish prey resources during the two periods (2010 and 2020) have prompted *S. tumbil* to diversify its prey, utilize alternative resources, and expand its foraging space, enabling adaptation to the changing ecological environment and sustaining population development. Similar to other generalist fishes, the ODSs of *S. tumbil* indicate a partial transition towards larger prey items. However, the timing and magnitude of the ODSs varied between the two periods, reflecting life-history variations and adaptive adjustments to environmental changes.

In Chinese waters, including the Beibu Gulf, the shift in the composition of dominant fishes from high-trophic-level demersal species to low-trophic-level pelagic species, coupled with a gradual decrease in the MTL, highlights that, in 2020, the *S. tumbil* population exhibited a significantly higher proportion of feeding on pelagic–neritic prey compared to 2010, despite a decrease in trophic levels. This suggests that the transition in the ecological type of the prey (from demersal to pelagic–neritic) is a primary factor contributing to the decrease in trophic levels.

Our future research will further integrate spatiotemporal data of both predators and prey in order to conduct quantitative analyses to elucidate the relationships between marine predator feeding behavior, nutritional levels, and changes in prey community structure.

## Figures and Tables

**Table 1:** Structure of *Saurida tumbil* specimens captured in the Beibu Gulf in 2010 and 2020. I–V represent the five size classes derived from body length (BL) measurements: I (BL < 14.1 cm), II (BL = 14.1–17.0 cm), III (BL = 17.1–20.0 cm), IV (BL = 20.1–23.0 cm), and V (BL > 23.0 cm).

Year	Size Class	Body Length (BL cm)	Individuals Sampled	Stomachs Analyzed	Vacuity Index (%)	Stable Isotopes Analysis
2010	I	<14.1	82	38	36.67	35
	II	14.1–17.0	221	97	38.99	42
	III	17.1–20.0	255	149	26.24	43
	IV	20.1–23.0	92	47	32.37	26
	V	>23.0	20	7	48.15	18
		Total specimens	670	338	49.55	164
2020	I	<14.1	36	21	26.32	23
	II	14.1–17.0	182	86	35.82	66
	III	17.1–20.0	266	130	34.34	54
	IV	20.1–23.0	166	78	36.07	43
	V	>23.0	58	21	46.84	24
		Total specimens	708	336	52.54	210

**Table 2:** Stomach specimens of *Saurida tumbil* captured in the Beibu Gulf in 2010 and 2020. BL (body length), GSI% (gonadosomatic index), RI% (relative index), and δ^15^N (nitrogen stable isotope values) were calculated, and within-group variance analysis and multiple comparisons among the size classes were carried out. I–V represent the five size classes based on body length (BL): I (BL < 14.1 cm), II (BL = 14.1–17.0 cm), III (BL = 17.1–20.0 cm), IV (BL = 20.1–23.0 cm), and V (BL > 23.0 cm). Different letters represent different significant levels. Size classes with the same letter do not exhibit a significant difference, while groups with different letters indicate a significant difference. Lowercase and Uppercase letters are used to label the specimens from 2010 and 2020, respectively.

Year	Size Class	BL Mean ± s.d.	GSI% Mean ± s.d.	RI% Mean ± s.d.	δ^15^N Mean ± s.d.
2010	I	12.03 ± 0.64	0.16 ± 0.10 ^d^	19.82 ± 9.82 ^c^	12.94 ± 1.31 ^b^
	II	15.23 ± 1.07	0.66 ± 1.16 ^cd^	14.54 ± 9.22 ^b^	13.52 ± 1.53 ^ab^
	III	18.18 ± 0.79	1.25 ± 1.99 ^bc^	10.09 ± 7.57 ^a^	13.74 ± 1.30 ^ab^
	IV	21.23 ± 0.80	2.65 ± 2.71 ^a^	13.57 ± 9.64 ^ab^	13.96 ± 1.08 ^a^
	V	24.80 ± 1.52	3.06 ± 2.93 ^ab^	10.55 ± 8.41 ^abc^	13.69 ± 1.11 ^ab^
2020	I	13.18 ± 0.79	0.10 ± 0.10 ^BC^	13.17 ± 4.63 ^AB^	12.44 ± 1.03 ^B^
	II	15.77 ± 0.85	0.33 ± 0.84 ^B^	10.36 ± 7.93 ^AB^	13.08 ± 0.91 ^A^
	III	18.52 ± 0.87	1.28 ± 1.71 ^C^	10.06 ± 10.18 ^A^	13.15 ± 0.98 ^A^
	IV	21.35 ± 0.86	2.78 ± 2.98 ^A^	13.98 ± 10.73 ^B^	13.43 ± 0.78 ^A^
	V	24.23 ± 0.93	4.00 ± 3.30 ^A^	11.71 ± 11.18 ^AB^	13.46 ± 0.76 ^A^

**Table 3:** The diet composition of the *Saurida tumbil* captured between 2010 and 2020 in the Beibu Gulf. F% (frequency of occurrence), W% (percentage in biomass), N% (percentage of number), IRI (index of relative importance), and IRI% (index of relative importance expressed as a percentage) values are shown to provide insight into the vertical habitat zones and behavior of each prey item. Vertical habitat zones: P—pelagic–neritic, D—demersal, and B—benthic; behavior: C—clustering, and M—migratory.

Taxa	Habitat	Behavior	2010					2020				
			N%	F%	W%	IRI	IRI%	N%	F%	W%	IRI	IRI%
Crustacea			5.96	3.53	1.61	26.77	0.17	3.22	3.80	0.96	15.88	0.12
Scyllaridae												
*Scyllarus* sp.	B		-	-	-	-	-	0.23	0.27	0.08	0.08	0.01
Penaeidae												
*Metapenaeopsis barbata*	B		1.65	1.90	1.08	5.19	0.34	0.23	0.27	0.03	0.07	0.01
*Metapenaeopsis acclivis*	B		-	-	-	-	-	0.46	0.54	0.24	0.38	0.03
*Trachypenaeus curvirostris*	B		0.95	1.09	0.48	1.55	0.10	0.69	0.82	0.11	0.66	0.06
Solenoceridae												
*Solenocera* sp.	B		-	-	-	-	-	0.46	0.54	0.04	0.27	0.02
*Solenocera crassicornis*	B		-	-	-	-	-	0.23	0.27	0.14	0.10	0.01
Squillidae												
*Oratosquilla*.sp.	B		0.47	0.54	0.06	0.29	0.02	0.69	0.82	0.12	0.66	0.06
*Oratosquilla oratoria*	B		-	-	-	-	-	0.23	0.27	0.19	0.11	0.01
Cephalopoda			7.57	8.42	12.13	165.97	1.08	11.95	13.59	32.75	607.43	4.63
Loliginidae												
*Loligo* sp.	P	C	5.20	5.71	5.63	61.81	4.04	6.90	7.88	15.82	179.04	15.23
*Uroteuthis chinensis*	P	C	2.36	2.45	6.34	21.30	1.39	2.30	2.45	7.02	22.79	1.94
*Uroteuthis duvauceli*	P	C	0.24	0.27	0.16	0.11	0.01	2.76	3.26	9.91	41.31	3.51
Fishes			86.47	88.04	86.25	15,206.96	98.75	84.83	82.61	66.29	12,483.70	95.24
Clupeidae												
*Sardinella jussieu*	P	C, M	4.26	4.35	7.44	50.84	3.32	0.23	0.27	0.29	0.14	0.01
*Sardinella lemuru*	P	C, M	-	-	-	-	-	3.68	4.35	12.40	69.91	5.95
Engraulidae												
*Stolephorus indicus*	P	C	7.57	7.34	5.19	93.60	6.12	5.52	3.53	3.35	31.32	2.67
*Stolephorus commersonnii*	P	C	0.24	0.27	0.04	0.08	0.00	1.38	1.36	0.65	2.76	0.23
*Stolephorus chinensis*	P	C	0.47	0.54	0.36	0.45	0.03	-	-	-	-	-
*Stolephorus* sp.	P		6.38	6.52	5.95	80.41	5.26	20.69	21.74	6.56	592.47	50.41
*Thryssa dussumieri*	P	C	0.95	1.09	0.43	1.49	0.10	3.22	3.80	2.78	22.80	1.94
*Thryssa setirostris*	P		-	-	-	-	-	0.23	0.27	0.37	0.16	0.01
*Thryssa* sp.	P		0.95	1.09	0.53	1.60	0.10	0.46	0.54	0.21	0.36	0.03
Bregmacerotida												
*Bregmaceros rarisquamosus*	P	C, M	1.42	1.63	0.99	3.93	0.26	5.75	3.26	0.89	21.64	1.84
*Bregmaceros mcclellandii*	P	C, M	-	-	-	-	-	0.23	0.27	0.19	0.11	0.01
*Bregmaceros* sp.	P		-	-	-	-	-	2.99	3.26	0.49	11.35	0.97
Leiognathidae												
*Photopectoralis bindus*	D	C	12.29	12.50	6.98	240.89	15.74	1.84	1.90	0.55	4.55	0.39
*Secutor ruconius*	D	C	-	-	-	-	-	5.06	5.43	4.27	50.69	4.31
*leiognathus lineolatus*	D	C	20.57	16.30	4.66	411.39	26.88	-	-	-	-	-
*Nuchequula nuchalis*	D	C	-	-	-	-	-	1.15	0.82	0.21	1.11	0.09
*Leiognathus berbis*	D	C	-	-	-	-	-	2.76	2.45	0.48	7.92	0.67
*Gazza minuta*	D		-	-	-	-	-	0.23	0.27	0.04	0.07	0.01
*Leiognathus* sp.	D		-	-	-	-	-	6.67	5.98	1.53	48.97	4.17
Acropomatidae												
*Acropoma japonicum*	D		7.80	7.88	6.91	115.93	7.58	3.22	2.72	1.60	13.10	1.11
Apogonidae												
*Ostorhinchus pleuron*	D		3.07	3.53	5.17	29.10	1.90	1.38	1.63	1.26	4.30	0.37
*Jaydia lineata*	D		0.24	0.27	0.09	0.09	0.01	0.23	0.27	0.37	0.16	0.01
*Ostorhinchus gularis*	D		-	-	-	-	-	0.46	0.54	0.19	0.36	0.03
*Jaydia carinatus*	D		-	-	-	-	-	0.46	0.54	0.97	0.78	0.07
*Rhabdamia gracilis*	D		-	-	-	-	-	0.23	0.27	0.07	0.08	0.01
*Jaydia striata*	D		0.47	0.54	0.80	0.69	0.05	0.23	0.27	0.05	0.08	0.01
*Jaydia poecilopterus*	D		0.24	0.27	0.28	0.14	0.01	1.15	1.36	1.24	3.24	0.28
Synodontidae												
*Saurida tumbil*	B		0.71	0.82	1.92	2.14	0.14	0.46	0.54	2.58	1.65	0.14
*Saurida* sp.	B		-	-	-	-	-	1.38	1.63	2.95	7.06	0.60
Carangidae												
*Decapterus maruadsi*	P	C, M	5.20	4.35	10.96	70.27	4.59	0.46	0.54	3.16	1.97	0.17
*Trachurus japonicus*	P	C	10.40	11.96	16.11	316.98	20.72	-	-	-	-	-
Siganidae												
*Siganus fuscescens*	D		-	-	-	-	-	0.23	0.27	0.67	0.25	0.02
*Siganus* sp.	D		0.47	0.54	1.12	0.87	0.06	0.23	0.27	0.68	0.25	0.02
Nemipteridae												
*Nemipterus nematophorus*	D		-	-	-	-	-	0.23	0.27	1.71	0.53	0.04
Mullidae												
*Upeneus sulphureus*	D		0.24	0.27	1.03	0.34	0.02	0.46	0.54	0.50	0.52	0.04
*Upeneus bensari*	D		0.24	0.27	1.59	0.50	0.03	0.46	0.27	0.28	0.20	0.02
Champsodontidae												
*Champsodon snyderi*	B		-	-	-	-	-	2.53	0.82	0.43	2.41	0.21
*Champsodon atridorsalis*	B		-	-	-	-	-	0.69	0.82	0.20	0.73	0.06
Trichiuridae												
*Trichiurus japonicus*	B		-	-	-	-	-	0.46	0.54	0.36	0.44	0.04
*Trichiurus* sp.	B		-	-	-	-	-	2.53	2.72	3.28	15.79	1.34
Sciaenidae												
*Pennahia pawak*	D		-	-	-	-	-	0.23	0.27	0.17	0.11	0.01
*Pennahia argentata*	D		-	-	-	-	-	0.46	0.54	0.33	0.43	0.04
*Johnius* sp.	D		-	-	-	-	-	0.46	0.54	0.77	0.67	0.06
*Pennahia* sp.	D		-	-	-	-	-	0.23	0.27	0.05	0.08	0.01
Sparidae												
*Evynnis cardinalis*	D		0.24	0.27	0.19	0.12	0.01	-	-	-	-	-
Gobiidae												
*Oxyurichthys tentacularis*	B		-	-	-	-	-	0.23	0.27	0.35	0.16	0.01
*Trypauchen vagina*	B		-	-	-	-	-	0.23	0.27	0.02	0.07	0.01
*Oxyurichthys* sp.	B		-	-	-	-	-	0.23	0.27	0.34	0.15	0.01
*Gobiidae* sp.	B		1.89	2.17	2.82	10.25	0.67	0.69	0.82	0.44	0.92	0.08
Citharidae												
*Brachypleura novaezeelandiae*	B		1.42	1.63	2.28	6.03	0.39	-	-	-	-	-
Triglidae												
*Pterygotrigla hemisticta*	B		0.24	0.27	0.12	0.10	0.01	-	-	-	-	-
Soleidae												
*Solea ovata*	B		0.47	0.54	1.12	0.86	0.06	-	-	-	-	-
Cynoglossidae												
*Cynoglossus* sp.	B		-	-	-	-	-	0.69	0.82	0.97	1.35	0.11
Callionymidae												
*Callionymidae* sp.	B		-	-	-	-	-	0.23	0.27	0.02	0.07	0.01
Bothidae												
*Arnoglossus* sp.	B		-	-	-	-	-	0.23	0.27	0.12	0.09	0.01
Tetraodontidae												
*Lagocephalus spadiceus*	B		-	-	-	-	-	0.46	0.54	2.02	1.35	0.11
Platycephalidae												
*Thysanophrys chiltonae*	B		0.47	0.54	0.72	0.65	0.04	-	-	-	-	-
Samaridae												
*Samaris cristatus*	B		0.24	0.27	0.47	0.19	0.01	-	-	-	-	-

**Table 4:** Indices for quantifying the trophic niche and individual specialization of *Saurida tumbil*. TNW represents the total niche width; WIC represents within-individual variations; BIC represents between-individual variations; WIC/TNW represents the degree of individual diet specialization; and *PS_i_* represents the proportional similarity index for a diet. I–V represent the five size classes based on body length (BL): I (BL < 14.1 cm), II (BL = 14.1–17.0 cm), III (BL = 17.1–20.0 cm), IV (BL = 20.1–23.0 cm), and V (BL > 23.0 cm).

	2010						2020					
Indices	Total Specimens	I	II	III	IV	V	Total Specimens	I	II	III	IV	V
WIC	0.107	0.068	0.066	0.115	0.162	0.252	0.117	0	0.026	0.094	0.224	0.187
BIC	2.630	2.225	2.455	2.384	2.599	1.516	3.151	0.730	2.340	3.193	2.662	2.414
TNW	2.737	2.293	2.521	2.498	2.761	1.768	3.267	0.730	2.366	3.287	2.887	2.602
WIC/TNW	0.039	0.029	0.026	0.046	0.059	0.143	0.036	0	0.011	0.029	0.078	0.072
*PS_i_*	0.117	0.166	0.127	0.163	0.115	0.361	0.088	0.649	0.211	0.076	0.117	0.138

## Data Availability

To obtain data from this study, please contact author Xiaodong Yang (email: yangxd2832@163.com).
